# A Reproducible COCO-Polygon Quality-Control Pipeline Improves Segmentation Stability in Endoscopic Airway Imaging

**DOI:** 10.3390/diagnostics16081160

**Published:** 2026-04-14

**Authors:** Medine Atmaca, Ilkay Sibel Kervancı, Necati Olgun

**Affiliations:** 1 Department of Mathematics, Graduate School of Natural and Applied Sciences, Gaziantep University, 27410 Gaziantep, Türkiye; 2 Department of Computer Engineering, Faculty of Engineering, Gaziantep University, 27410 Gaziantep, Türkiye; skervanci@gantep.edu.tr

**Keywords:** endoscopy, airway management, deep learning, image segmentation, reproducibility of results

## Abstract

**Background/Objectives**: Endoscopic airway imaging, used in endoscopy-guided airway management, enables geometry-based assessment of the airways. However, COCO-format datasets often include fragmented regions and geometrically inconsistent polygon annotations. Such inconsistencies may reduce reproducibility and spatial stability in deep learning-based image segmentation. This study proposes a systematic annotation quality-control (QC) workflow to improve dataset integrity before model training. **Methods**: The phantom subset of the Upper Airway Anatomical Landmark (UAAL) dataset containing 4526 polygon instances across 2746 frames (2267 training; 479 validation) was analyzed. The QC pipeline validated polygon structure, generated masks at native image resolution, and removed small noise-like instances using an area threshold. A YOLOv8-seg model was trained using (i) original annotations and (ii) QC-refined annotations. Performance was evaluated using precision, recall, mAP@0.5, mAP@0.5:0.95, and Dice similarity coefficient (DSC). Frame-level DSC values were compared using the Wilcoxon signed-rank test. **Results**: Annotation QC improved boundary consistency and reduced mask fragmentation. Training with QC-refined annotations increased box mAP@0.5:0.95 from 0.602 to 0.628 and mean DSC from 0.823 to 0.830 (*p* < 0.05). A pilot evaluation on the UAAL clinical subset also showed improved performance, with Box mAP@0.5 increasing from 0.635 to 0.706 and Mask mAP@0.5 from 0.631 to 0.704. **Conclusions**: Annotation-level QC enhances segmentation robustness without modifying the network architecture. The proposed workflow improves the reproducibility of results in endoscopic image segmentation and may support more stable geometry-based airway analysis in deep learning applications.

## 1. Introduction

Difficult laryngoscopy and endotracheal intubation continue to present significant challenges and are associated with increased morbidity and procedural complications [1]. Bedside assessment tools often show limited predictive performance, particularly in unanticipated difficult airway scenarios [2]. Airway imaging has progressed significantly in recent years, particularly in the context of flexible endoscopy and videolaryngoscopy, allowing direct visualization of upper airway anatomy during airway management.

Previous studies indicate that, in deep learning-based medical image analysis, annotation quality may influence segmentation performance more strongly than annotation quantity [3]. Systematic dataset curation and high-quality annotations have been determined to be critical in establishing reliable and reproducible deep learning models in medical imaging [4]. Convolutional neural networks have shown strong performance in medical image segmentation tasks and have been proposed for objective airway assessment [5]. Recent reviews confirm that the role of artificial intelligence in airway management is increasing, including for decision support and integration with endoscopic imaging systems, though AI pipelines need to be reproducible and reliable before clinical application [6]. To this end, we developed an instance segmentation workflow for endoscopic airway images using YOLOv8-seg together with a quality-controlled COCO annotation pipeline. YOLOv8-seg was selected for its stable training behavior and transparent implementation in medical imaging tasks [7]. Recent clinical research also indicates that YOLO-based instance segmentation can delineate airway-related anatomical structures such as laryngeal regions in real medical images [8].

Real-world challenges in airway endoscopy, including blood contamination, motion blur, occlusions, and variable illumination, may lead to inconsistent or noise-like polygon annotations, affecting segmentation stability. Semi-automated annotation quality control systems emphasize the need for end-to-end validation pipelines to enhance dataset consistency and model robustness [9]. Annotation-efficient deep learning toolkits similarly highlight the importance of quality-aware dataset preparation when dealing with limited or noisy labels [10].

When label noise and annotation quality are modelled in training, they directly impact deep learning performance [11]. This is particularly relevant in medical imaging, where annotation errors can propagate into systematic model biases. Learning from multiple annotators and accounting for inter-annotator variability may improve robustness and reliability in segmentation tasks [12]. Model performance in biomedical image segmentation heavily relies upon label consistency and annotation reliability [13], reinforcing the need for structured quality assurance before model training. Methods addressing label noise and incomplete supervision have recently attracted increasing attention [14], reflecting a broader shift toward data-centric approaches in deep learning. Annotation noise can hinder generalizability and segmentation accuracy [15], and refinement approaches based on active learning and collaborative learning have been proposed to address annotation efficiency and label scarcity [16,17]. These findings collectively motivate the structured QC approach proposed in the present study. Although the COCO annotation format has become ubiquitous for instance segmentation, raw COCO annotations typically need to be validated and quality controlled before reliable model development begins [18].

Polygon annotations were first validated and converted to binary masks at native image resolution, followed by suppression of small noise-like instances based on an area-based criterion. Performance was evaluated at both qualitative and quantitative levels, including frame-level Dice similarity coefficient (DSC) values to assess boundary stability. Such an assessment is especially important for new datasets that have not yet undergone structured quality-control evaluations [19]. The UAAL dataset (September 2025) is a recently released upper-airway resource that has not yet been explored within a structured COCO-polygon quality-control framework. Overall, this research is one of the first to quantify how polygon-level refinement impacts segmentation stability in UAAL, to the best of our knowledge. While prior work has addressed annotation quality primarily through semantic label noise correction [20], these approaches do not target the structural validity of polygon geometries. Annotation management platforms offer interface-level review mechanisms, yet they do not provide domain-specific, quantifiable QC metrics tailored to COCO-polygon instance segmentation datasets. The proposed pipeline addresses a complementary and distinct problem: geometric annotation integrity, specifically targeting invalid polygon structures, native-resolution mask rasterization, and area-threshold-based noise filtering, properties that general-purpose dataset cleaning methods do not systematically handle. Similar studies have emphasized that inconsistencies in annotations may influence segmentation performance and therefore require dedicated inspection mechanisms [21].

## 2. Materials and Methods

### 2.1. Dataset Characteristics

The dataset analyzed in this study was adapted from the publicly available Upper Airway Anatomical Landmark (UAAL) dataset (Version 4, Figshare, 2025) [22]. A total of 3814 clinical endoscopic images (from 82 patients) and 2746 phantom images were collected as part of the UAAL dataset. In total, the clinical subset consists of 10,330 annotations (4910 instance segmentation masks and 5420 bounding boxes) in eight anatomical classes, and the phantom subset consists of 4526 annotations in nine classes.

In this research, the phantom subset was used mainly to investigate the impact of annotation quality control in the controlled imaging study setting. It gives polygon-based instance segmentation annotations of airway landmarks from the nasal cavity to the trachea. The subset consists of 2746 annotated frames divided into 2267 training images and 479 validation images. All analyses were done at a frame level.

The phantom subset was chosen based on the concern of minimizing confounding influences (secretion, bleeding, motion artifacts, extreme illumination alterations). This approach also facilitated a more direct analysis of the impact of annotation quality control on segmentation stability. Invalid polygon entries and small spurious masks were removed prior to model training to increase the uniformity and repeatability of the annotation during the QC process.

Real world transferability was estimated by performing a pilot study on the UAAL clinical subset with the same QC technique and YOLOv8-seg training set up. Performance was examined using the clinical validation split. This pilot trial was aimed at proving it to be realistic with realistic endoscopic variability as opposed to establishing a clinical reference point.

### 2.2. Overview of the Annotation and Quality-Control Pipeline

For deep learning model development to provide reliable ground truth, structured annotation workflows and expert validation for large-scale clinical imaging data are required [23]. Clinical knowledge is especially crucial in annotation design, as well as iterative quality analysis, to ensure dataset reliability in medical imaging tasks [24].

Airway annotations collected via COCO format were preprocessed to obtain a training dataset with validated polygons and cleaned instance masks. The pipeline provides mask generation to be standardized, invalid geometries are removed, and small fragmented instances are filtered before model training. Weakly supervised learning techniques have been suggested to alleviate manual annotation effort in medical image segmentation [25].

The annotation workflow starts with parsing JSON annotation files in COCO format followed by polygon validation. Invalid polygon entries are identified and processed safely to avoid errors during mask generation. Verified polygon entries are converted to instance-level binary masks.

Mask filtering based on area is used to eliminate noise-like or clinically irrelevant annotations. Subsequent analysis excludes any instances which are less than the defined pixel-area threshold. It then produces overlay-based visualizations, showing filtered masks placed over original endoscopic frames to monitor their quality for visual inspection. In addition, overlay visualizations can inspect annotation quality for spatial consistency manually in real time. The results consist of clean masks and visualization-ready pictures, which make up the standardized dataset. Automated or AI-assisted annotation workflows should be validated against expert-reviewed labels to ensure reliability and reproducibility [26].

**Figure 1:**
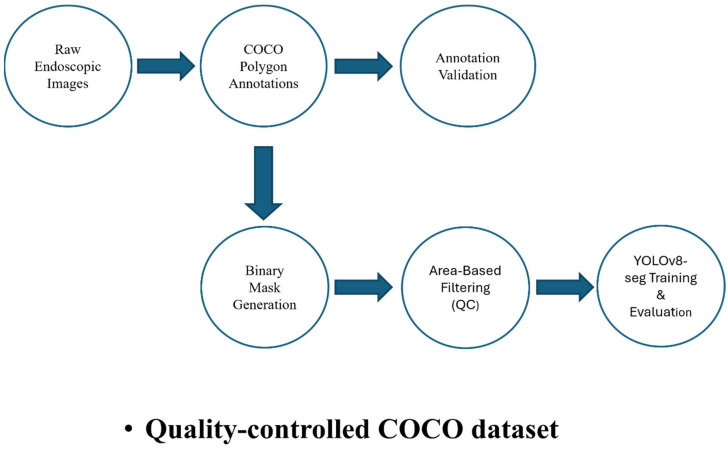
Overview of the proposed annotation QC and segmentation pipeline. Raw endoscopic images and COCO-style polygon annotations undergo validation, binary mask generation, and area-based filtering to produce a quality-controlled dataset used for YOLOv8-based instance segmentation training and evaluation.

An overview of the proposed annotation QC and segmentation workflow is illustrated in Figure 1.

### 2.3. COCO Annotation Format and Dataset Structure

The dataset used in this study uses the COCO instance-segmentation format. Our QC pipeline supports polygon validation, mask rasterization, and area-based filtering by using the JSON-based structure. All JSON files present image metadata (such as the ID, file name, width, and height) and annotation entries that specify image IDs, category labels, and polygon segmentation coordinates. Polygon annotations are defined with ordered (x, y) vertex coordinates specifying airway landmarks; there may be several instance polygons in one image. Category definitions are stored independently and referred to by annotations for consistent class IDs. Overall, this structure allows us to parse the data accurately for polygon validation and native-resolution mask generation in our QC workflow [27].

### 2.4. Annotation Validation and Binary Mask Generation

**Figure 2:**
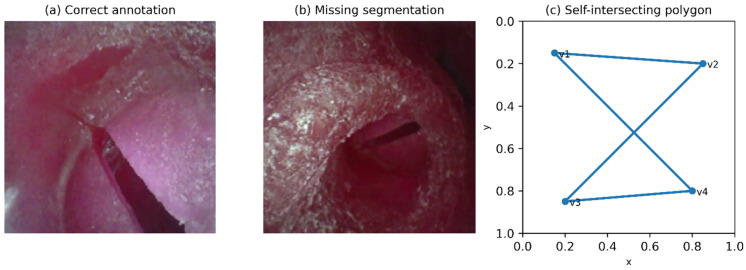
Representative examples of annotation quality and invalid polygon geometries. (**a**) Correct annotation with accurate boundary alignment. (**b**) Missing segmentation example. (**c**) Example of a self-intersecting polygon illustrating an invalid geometry addressed during the QC process.

Polygon annotations were screened for structural and geometric validity before mask generation [28]. Entries with missing/empty segmentation, fewer than three vertices, out-of-bounds coordinates, or degenerate shapes yielding near-zero area were excluded. The remaining polygons were rasterized into binary masks at the native image resolution to maintain pixel-level alignment between images and annotations. Masks were created separately for each instance of frames containing multiple instances and used in later filtering, visualization, and sequence-level analyses. Representative examples of annotation quality variations and invalid polygon geometries are illustrated in Figure 2.

### 2.5. Area-Based Filtering and Noise Suppression

After binary mask generation, an area-based filtering method was applied to filter against noise-like or clinically irrelevant annotation instances [29]. Similar post-processing strategies, including small-object removal and morphological operations, have also been incorporated into recent medical image segmentation toolkits to refine segmentation outputs [30]. In this context, small annotation regions could surface due to boundary-related artifacts and labeling inconsistencies which can limit the overall dataset quality and affect downstream processing [31]. Detection-guided and area-based post-processing techniques are broadly used in medical image segmentation pipelines to detect and eliminate false small areas for robustness before performing further analyses [32]. Annotated pixel area was calculated for each binary mask to evaluate how the filtering is performing through area methods. Masks with a pixel area below the pre-set minimum threshold were removed from further processing. The filtering step is threshold-based in order to remove noise-like annotations and maintain clinically relevant anatomical regions.

The filtering strategy applied is to the entire dataset to ensure consistency and reproducibility. The suppression of small patches and fragmented content in this filtering step results in greater dataset robustness, thereby creating a more reliable geometric analysis and training data in subsequent work phases.

### 2.6. Sensitivity Analysis for Area Threshold Selection

Multiple candidate threshold settings were checked for their effect on annotation retention. For all examined thresholds, exclusion rates and visual annotation features were analyzed using structured qualitative assessment. Thresholds with high removal of clinically relevant regions were considered too constraining. Noise suppression was regarded as not optimal for thresholds that preserved many small or fragmented regions.

**Figure 3:**
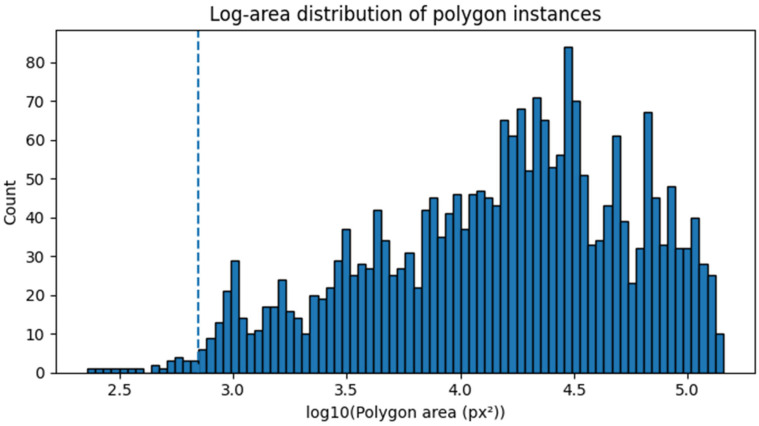
Log-scale distribution of polygon instance areas in the UAAL phantom dataset. The distribution exhibits a strongly right-skewed pattern with numerous small fragmented annotations. The dashed vertical line indicates the selected minimum-area threshold (700 px^2^) used for annotation filtering.

With an extensive qualitative and quantitative analysis, we established a threshold which was to preserve the anatomical integrity but suppress the noise-like regions. A thorough analysis of area distribution revealed significant right-skewed distribution (median: 17,879 px^2^; mean: 28,896 px^2^) with small fragmented instances. The distribution of polygon areas is illustrated in Figure 3. The area statistics on a training split (*n* = 2267 images) from all cases with valid polygons prior to area-based filtering (*n* = 2370 polygons) were calculated. The 2nd percentile was about 879 px^2^. We established a minimum area threshold of 700 px^2^ to reduce noise-like regions and retain anatomically meaningful structures by way of distributional inspection and overlay-based qualitative assessment. Only a few instances were close to a zero area, suggesting degenerate or malformed polygons eliminated by polygon validation. This threshold was set prior to the model evaluation and was not designed to facilitate segmentation performance with optimized performance. This threshold was selected prior to model training to prevent post hoc optimization and to maintain a data-driven threshold selection procedure.

The sensitivity analysis of the minimum-area threshold (Table 1) shows that lower thresholds (300–500 px^2^) have a negligible filtering effect, while higher thresholds (1000 px^2^) lead to aggressive removal of annotations. A threshold of 700 px^2^ provides the best trade-off between noise suppression and preservation of valid anatomical structures.

### 2.7. Overlay Visualization and Manual Quality Assessment

Regarding qualitative evaluation of annotation quality, the proposed pipeline generates semi-transparent overlay visualizations of filtered binary masks overlaid on the original endoscopic airway images. This overlay visualization method makes visualization of the annotations easy in terms of alignment, boundary fidelity, and anatomical plausibility [33].

Similar high-resolution overlays with consistent transparency and color settings were prepared for ease of visual comparison from one sample to another. Based on these overlay visualizations, annotators and researchers are able to quickly identify labeling artefacts, misaligned segmentation, or omitted sections that otherwise may not be apparent by means of numerical metrics alone.

Overlay visualizations serve as a practical error detection mechanism at three levels. First, misaligned masks where the colored overlay does not coincide with the anatomical structure boundary indicate polygon coordinate errors or resolution mismatches during mask rasterization. Second, isolated small overlay regions appearing outside the main anatomical area are indicative of noise-like or spurious annotations that may survive polygon validation but are subsequently removed by area-based filtering. Third, fragmented overlays covering discontinuous regions within a single instance reveal structurally invalid geometries. By systematically reviewing these overlay reports before model training, annotation errors that are not detectable through numerical metrics alone can be identified and addressed, improving the overall integrity of the training dataset.

**Figure 4:**
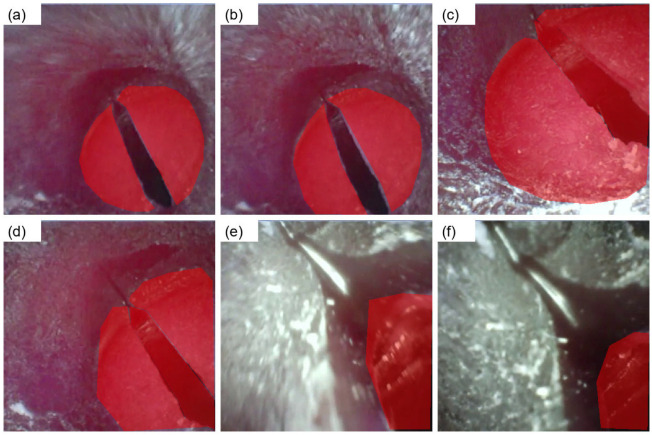
Overlay visualization of binary segmentation masks on endoscopic upper-airway images from the UAAL phantom subset. The red semi-transparent overlay indicates the QC-refined binary mask for each frame. Well-aligned masks closely follow anatomical boundaries, while misaligned or fragmented overlays appearing outside the primary anatomical region indicate noise-like annotations targeted for removal by area-based filtering. Panels (**a**–**f**) show six representative frames illustrating viewpoint and anatomical variability across the dataset.

**Figure 5:**
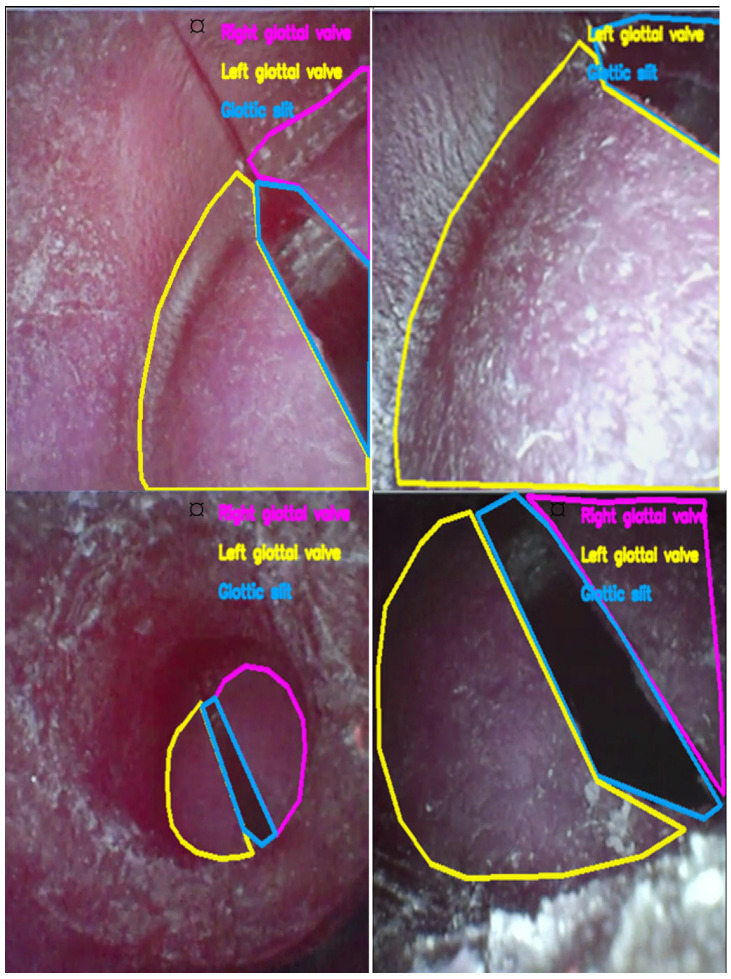
Representative examples of manually annotated airway structures across different endoscopic frames from the UAAL phantom subset. Colored contours denote distinct anatomical classes: each color corresponds to a separate annotation instance. Consistent color coding across panels allows visual comparison of annotation coverage and boundary fidelity across frames.

This inspection is done manually and provides a second layer of validation after the filter applied to automate processes. As the quality of airway imaging data can be analyzed with quantitative filtering in conjunction with visual inspection using qualitative methods, this encourages the construction of a robust analysis-ready dataset for sequence-level geometric analysis and additional model formulation. Graphical examples of overlay visualization are provided in Figure 4, and manual polygon annotations are shown in Figure 5 as representative examples.

### 2.8. Computing Environment (Computational Resources)

All experiments were conducted in Google Colab using an NVIDIA Tesla T4 GPU (15 GB VRAM). The software environment included Python 3.12.13, PyTorch 2.10.0+cu128, Ultralytics YOLOv8 8.4.31, and OpenCV 4.13.0. Polygon validation, mask rasterization, and area-based filtering were implemented using Python’s standard JSON parsing libraries in conjunction with OpenCV for mask generation and NumPy for array operations.

## 3. Results

### 3.1. Dataset Cleaning and Annotation Refinement Outcomes

We removed annotations with missing segmentation fields, structurally invalid polygons, and masks below the predefined pixel-area threshold. After QC, overlay inspection showed fewer fragmented regions and improved boundary coherence across frames.

Polygon validation eliminated 1551 instances, and area-based filtering removed 31 additional masks (area < 700 px^2^), reducing the total annotations from 4526 to 2944 (Table 2). In the validation split, 256 invalid annotations and 8 small masks were excluded, leaving 597 retained annotations.

The final curated dataset consisted of standardized, analysis-ready images and corresponding masks suitable for sequence-level geometric analysis and subsequent model development. A quantitative overview of the curated dataset and annotation refinement process is reported in Table 2.

The final curated dataset consisted of standardized, analysis-ready images and corresponding masks suitable for sequence-level geometric analysis and subsequent model development.

### 3.2. Visualization Examples and Qualitative Results

The curated COCO-formatted dataset was mapped into a training-ready format for a YOLOv8 instance segmentation model after annotation validation and quality control. Binary masks created from validated polygon annotations were used to generate region-of-interest labels for each image in the dataset.

The dataset was split into training and validation sets at the image level to avoid information leakage across splits and to support robust evaluation [34]. All images and corresponding labels were scaled to a fixed input resolution compatible with YOLOv8 while preserving aspect ratio. The prepared dataset structure adhered to the conventional YOLO segmentation format, including distinct image and label directories and a configuration file defining classes and dataset paths.

### 3.3. YOLOv8-Seg Architecture

All experiments were performed with the Ultralytics YOLOv8 framework (Ultralytics, YOLOv8 version 8.x). The instance segmentation was performed with the YOLOv8-seg model that is a complementary construct to the YOLOv8 model, which uses a segmentation head for pixel-wise mask prediction. A convolutional backbone for feature extraction, a feature pyramid network for multi-scale representation, and a segmentation head that predicts object masks alongside bounding boxes and class probabilities are implemented in the implemented model.

Pretrained weights from large-scale image datasets were used to initialize model parameters when training on domain-specific medical images with limited sample sizes. The transfer-learning system allows for fast convergence and enhanced generalization. Despite the ongoing emergence of new YOLO variants, this study selected YOLOv8-seg as a stable and well-documented baseline for reproducible instance segmentation, while limiting confounding variability due to rapidly evolving model implementations. The following methodological choice corresponds to the methodological concern of the current study in quantifying the impact of dataset quality control. The YOLOv8-seg was chosen mainly due to its training stability, reproducibility, and widespread adoption in medical imaging research rather than architectural novelty.

### 3.4. Training Protocol

The model was trained for 100 epochs with a batch size of 16 at 640 × 640 resolution using a YOLOv8s-seg model initialized with pretrained weights (yolov8s-seg.pt). An initial learning rate of lr0 = 0.01 was used with automatic learning rate scheduling via the Ultralytics built-in cosine decay mechanism. The optimizer was set to auto (Ultralytics default) with momentum = 0.937 and weight decay = 5 × 10^−4^. Standard data augmentation strategies were applied during training, including mosaic augmentation, horizontal and vertical flipping, and HSV-based color jittering. Deterministic settings were applied with seed = 0 and AMP enabled. No explicit early stopping was applied; training was completed for the full 100 epochs to ensure a fair and consistent comparison between the unfiltered and QC-refined conditions. Validation was performed only on the held-out validation split.

### 3.5. Evaluation Metrics

Model performance was assessed through the YOLOv8 validation pipeline (precision, recall, mAP@0.5, and mAP@0.5:0.95) for the output of both box and mask. Dice similarity coefficient (DSC) was post hoc computed from pixel-level overlap between predicted and reference masks. Frame-level DSC was computed on the validation split (*n* = 479), and paired comparisons between the QC-trained and unfiltered-trained models were assessed using the Wilcoxon signed-rank test.

Detection and segmentation metrics on the validation set are summarized in Table 3 and Table 4, respectively. A direct comparison between the unfiltered and QC-refined training conditions is reported in Table 5.

### 3.6. Pilot Clinical Evaluation (UAAL Clinical Subset)

A pilot evaluation was performed on the UAAL clinical subset to assess the effectiveness of the proposed COCO-polygon QC pipeline under real endoscopic imaging conditions. Using the QC-refined clinical dataset and the same YOLOv8-seg training protocol, the validation performance of the QC-trained model reached Box mAP@0.5 = 0.706 and Box mAP@0.5:0.95 = 0.441, while the model trained on the unfiltered dataset achieved 0.635 and 0.379, respectively.

Similarly, mask-based evaluation demonstrated consistent improvements after applying the QC pipeline. Mask mAP@0.5 increased from 0.631 to 0.704, and Mask mAP@0.5:0.95 improved from 0.352 to 0.402 (Table 6).

Overall clinical performance remained lower than the phantom results due to increased variability, motion blur, illumination changes, and secretion-related occlusions commonly observed in real endoscopic imaging. Nevertheless, these results demonstrate that the proposed QC pipeline improves segmentation consistency not only in controlled phantom datasets but also in real clinical airway images. To ensure methodological consistency, the same preprocessing steps and training configuration were applied to both the unfiltered and QC-refined clinical datasets.

## 4. Discussion

This study demonstrated that polygon annotation integrity can serve as an experimentally controllable variable affecting segmentation stability [35,36]. Rather than pursuing architectural modifications, annotation structure was treated as a primary source of variability. Our experiments show that QC-based polygon refinement improves high-IoU performance and frame-level DSC stability while the underlying model remains unchanged, underscoring dataset engineering as an active design consideration rather than a static preprocessing step.

A slight decrease in Mask mAP@0.5 (0.877 → 0.872) was observed after QC refinement. This is attributable to the removal of small or fragmented annotations that may still contribute positively under the lenient IoU threshold of 0.50 despite suboptimal spatial alignment. In contrast, higher-IoU metrics benefit from improved annotation consistency, indicating that the QC pipeline favors spatial accuracy over coarse localization, which is particularly relevant for geometry-based airway analysis where boundary precision directly affects downstream measurements.

Annotation noise can reduce not only raw performance but also the reliability of evaluation metrics, particularly when errors are systematic [37]. Polygon validation, native-resolution mask rasterization, and area-based filtering reduced this bias at the annotation level. Overlay-based inspection confirmed increases in spatial coherence and anatomical plausibility, which is crucial in the upper airway where fragmented masks can compromise downstream geometric measurements.

In addition to missing or incomplete annotations, structurally invalid polygon geometries, such as degenerate shapes, fragmented regions, and self-intersecting polygons, were also identified and removed during the QC process.

Endoscopic airway images are inherently dynamic and segmentation results are rarely used in isolation [38]. Unstable masks across consecutive frames may compromise temporal and geometry-based assessment. The QC workflow suppresses noise-like regions and maintains valid polygon structures, leading to more stable anatomical boundaries across frames.

Clinical airway endoscopy introduces additional challenges including motion blur, variable illumination, and secretion-related occlusions. The phantom subset was employed as a controlled setting to isolate the impact of annotation quality control. A pilot evaluation on the UAAL clinical subset confirmed transferability, with the QC-refined model achieving Box mAP@0.5 = 0.706 and Mask mAP@0.5 = 0.704, compared to 0.635 and 0.631 for the unfiltered model. Absolute clinical metrics were lower than phantom results due to increased imaging variability, as expected in a feasibility assessment rather than a benchmark study.

While the numerical DSC difference may appear modest, small boundary variations in airway endoscopy can accumulate into larger deviations in longitudinal or geometry-based measurements.

These results indicate that structured dataset preparation influences segmentation stability in airway imaging. A transparent QC pipeline for COCO-style annotations provides a practical basis for sequence-level analysis and future geometry-based airway assessment.

Beyond airway endoscopy, the proposed QC pipeline may offer broader utility in imaging-driven clinical applications where annotation quality directly affects diagnostic reliability. Recent advances in virtual autopsy, which integrates CT, MRI, and ultrasound for non-invasive postmortem evaluation, represent one such emerging domain where robust segmentation and structured annotation validation may play an increasingly important role [39].

## 5. Study Limitations and Future Practical Implications

Several limitations of this study should be recognized. First, experiments were conducted on a single dataset (UAAL phantom subset), which limits the generalizability of findings to other airway imaging datasets or clinical settings. Second, a single segmentation architecture (YOLOv8-seg) was employed; whether the observed QC-induced improvements are fully model-agnostic remains to be confirmed across alternative architectures such as U-Net or Mask R-CNN. Third, the area threshold of 700 px^2^ was selected through distributional inspection rather than task-specific optimization, and may require recalibration for datasets with different annotation characteristics. Fourth, inter-annotator variability was not explicitly analyzed; in multi-center datasets with multiple annotators, systematic labeling inconsistencies beyond geometric errors may affect pipeline performance. Fifth, no temporal smoothing evaluation was performed; while the QC workflow improves frame-level mask consistency, its effect on temporal stability across consecutive frames has not been formally quantified. The proposed QC pipeline addresses structural polygon validity rather than semantic correctness; future work could combine geometric QC with semantic label quality estimation for a more comprehensive framework. Active learning techniques might further improve annotation efficiency [40], and uncertainty-aware training procedures could improve robustness under noisy annotations [41].

Future work should focus on validating the proposed pipeline across multiple datasets and segmentation architectures to confirm model-agnostic generalizability. Multi-center dataset validation would provide stronger evidence of transferability across different endoscopic systems, patient populations, and annotation workflows. Active learning strategies could further improve annotation efficiency by prioritizing ambiguous or low-quality samples for expert review, reducing the manual annotation burden in large-scale clinical datasets. Integration of the QC pipeline into clinical annotation workflows, potentially as an automated pre-training validation step within existing labeling platforms, represents a practical direction for real-world deployment. Quantitative threshold optimization, incorporation of inter-annotator variability analysis, and temporal smoothing evaluation represent additional natural extensions of this work. Beyond airway imaging, the pipeline may offer utility in other annotation-dependent clinical imaging domains where geometric annotation integrity directly affects downstream diagnostic reliability.

## 6. Conclusions

A transparent and reproducible QC workflow for COCO-style airway annotations is presented in the study, allowing improved dataset reliability and segmentation stability without architectural modification. Focusing on polygon validation, generation of native-resolution masks, and structured area-based filtering, the proposed pipeline enhances the boundary coherence and sequence-level consistency in endoscopic airway imaging.

These results indicate that annotation-level refinement may affect downstream segmentation robustness and evaluation stability. Careful dataset preparation should accordingly constitute a critical design component for medical image segmentation workflows, rather than a secondary preprocessing step. The proposed QC strategy may support future geometry-based airway assessment systems and AI-assisted clinical decision-support applications.

## Figures and Tables

**Table 1:** Sensitivity analysis of the minimum-area threshold.

Threshold (px^2^)	Removed Annotations	Retained Annotations	Removed (%)	Interpretation
300	3	3662	0.08%	Very permissive, almost no filtering
500	9	3656	0.25%	Slight filtering effect
700	23	3642	0.63%	Balanced noise removal and data retention
1000	77	3588	2.10%	Aggressive filtering, risk of removing valid regions

**Table 2:** Dataset summary and annotation refinement statistics after the proposed QC pipeline.

Metric	Value
Total frames	2746
Training frames	2267
Validation frames	479
Total annotations (raw)	4526
Removed invalid polygons	1551
Removed annotations (area < 700 px^2^)	31
Total removed (QC)	1582
Final annotations after QC	2944

**Table 3:** Detection-based performance metrics of the proposed YOLOv8-seg model on the validation set.

Metric	Value
Box Precision	0.807
Box Recall	0.873
Box mAP@0.5	0.874
Box mAP@0.5:0.95	0.628

**Table 4:** Mask-based segmentation performance metrics on the validation set.

Metric	Value
Mask Precision	0.814
Mask Recall	0.873
Mask mAP@0.5	0.872
Mask mAP@0.5:0.95	0.578
DSC	0.830

**Table 5:** Comparative performance between models trained on unfiltered annotations and quality-controlled annotations.

Metric	Unfiltered Dataset	QC Dataset (Proposed)
Box Precision	0.804	0.807
Box Recall	0.886	0.873
Box mAP@0.5	0.878	0.874
Box mAP@0.5:0.95	0.602	0.628
Mask mAP@0.5	0.877	0.872
Mask mAP@0.5:0.95	0.578	0.578
DSC	0.823	0.830

**Table 6:** Comparative performance between models trained on unfiltered and QC-refined clinical annotations.

Metric	Clinical Unfiltered	Clinical QC
Box mAP@0.5	0.635	0.706
Box mAP@0.5:0.95	0.379	0.441
Mask mAP@0.5	0.631	0.704
Mask mAP@0.5:0.95	0.352	0.402

## Data Availability

The UAAL dataset is publicly available on Figshare (Version 4, 2025) at https://doi.org/10.6084/m9.figshare.26342779 (accessed on 3 March 2026).
